# The vanishing atrial mass

**DOI:** 10.1093/ehjci/jew127

**Published:** 2016-07-07

**Authors:** Jason M. Tarkin, Deepa Gopalan, Mark R. Belham, James H.F. Rudd, Martin R. Bennett

**Affiliations:** 1 Division of Cardiovascular Medicine, University of Cambridge, Box 110, ACCI, Addenbrooke's Hospital, Cambridge CB2 2QQ, UK; 2 Department of Radiology, Cambridge University Hospitals NHS Trust, Cambridge, UK; 3 Department of Radiology, Imperial College Healthcare NHS Trust, London, UK; 4 Division of Cardiovascular Medicine, Cambridge University Hospitals NHS trust, Cambridge, UK

A 52-year-old woman with atopic asthma and a long smoking habit underwent CT pulmonary angiography (CTPA) for investigation of atypical, pleuritic chest pain. There was no history of heart disease or cardiovascular risk factors. As per usual practice for non-cardiac imaging, CTPA was performed without ECG gating. While there was no pulmonary embolism, CT showed an apparent large left atrial filling defect (arrows), demonstrated in axial (*Panel A*) and sagittal (*Panel B*) views. This CT abnormality raised the suspicion of left atrial thrombus or tumour, prompting further cardiac imaging.

Transoesophageal echocardiography, shown in short-axis (*Panel C*; see Supplementary data online, *Movie S1*) and four-chamber (*Panel D*; see Supplementary data online, *Movie S2*) views, showed the left atria to be of normal size and devoid of any visible mass. There was, however, an atrial septum aneurysm, which exhibited 2 cm shift from midline (arrows). Bi-ventricular size and function were normal, and there was no intra-atrial shunt seen on colour Doppler. No medical or surgical intervention was required for this incidental radiographic finding.

This report highlights a potential hazard of imaging the heart with non-ECG-gated CT, which is common practice when non-cardiac chest pain is suspected. Owing to aneurysmal septal motion, an unusual ‘ghost’ artifact appeared on this patient's CTPA, mimicking a left atrial mass. Diagnostic uncertainly led to unnecessary invasive testing. This case provokes the question: should ECG gating be added routinely when CTPA is performed for undifferentiated chest pain? (Ao, aorta; LA, left atrium; LV, left ventricle; PA, pulmonary artery; RA, right atrium).

**Figure:**
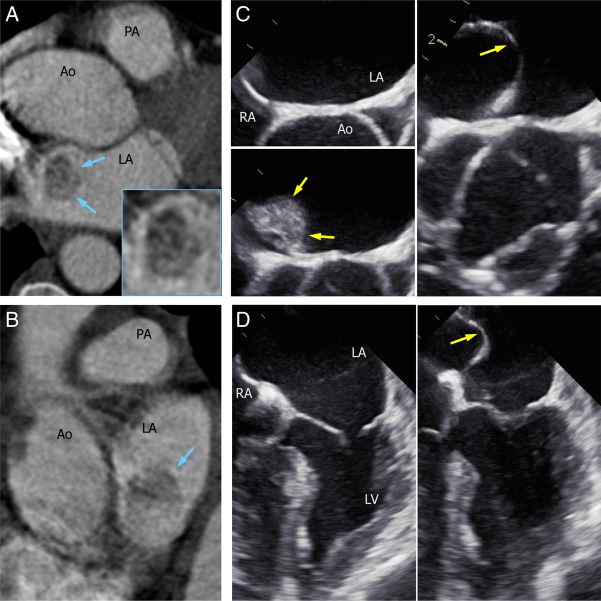

J.M.T. is supported by a Wellcome Trust research training fellowship (104492/Z/14/Z). J.H.F.R. is part supported by HEFCE. M.R.B. and J.H.F.R are supported by the NIHR Cambridge Biomedical Research Centre and the British Heart Foundation.

Supplementary data are available at *European Heart Journal— Cardiovascular Imaging* online.

